# Calcium-dependent electrostatic control of anion access to the pore of the calcium-activated chloride channel TMEM16A

**DOI:** 10.7554/eLife.39122

**Published:** 2018-10-12

**Authors:** Andy KM Lam, Raimund Dutzler

**Affiliations:** 1 Department of BiochemistryUniversity of ZurichZurichSwitzerland; 2 Department of BiochemistryUniversity of ZurichZurichSwitzerland; University of Wisconsin-MadisonUnited States; The University of Texas at AustinUnited States

**Keywords:** anoctamins, electrostatics, ion conduction, electrophysiology, ligand-gated ion channel, Mouse

## Abstract

TMEM16A is a ligand-gated anion channel that is activated by intracellular Ca^2+^. This channel comprises two independent pores and closely apposed Ca^2+^ binding sites that are contained within each subunit of a homodimeric protein. Previously we characterized the influence of positively charged pore-lining residues on anion conduction (Paulino et al., 2017a). Here, we demonstrate the electrostatic control of permeation by the bound calcium ions in mouse TMEM16A using electrophysiology and Poisson-Boltzmann calculations. The currents of constitutively active mutants lose their outward rectification as a function of Ca^2+^ concentration due to the alleviation of energy barriers for anion conduction. This phenomenon originates from Coulombic interactions between the bound Ca^2+^ and permeating anions and thus demonstrates that an electrostatic gate imposed by the vacant binding site present in the sterically open pore, is released by Ca^2+^ binding to enable an otherwise sub-conductive pore to conduct with full capacity.

## Introduction

**Figure 1.**
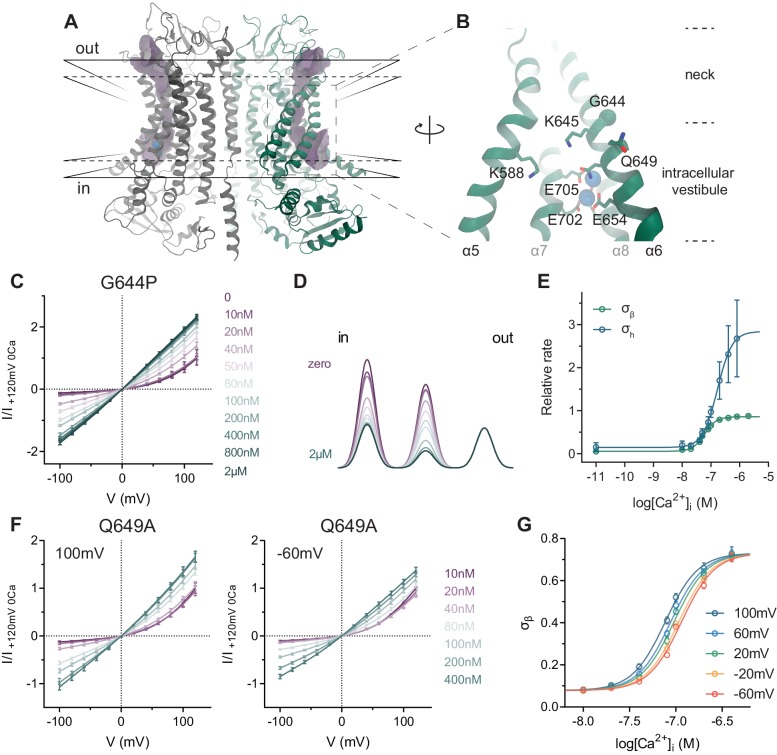
TMEM16A structure and conduction properties of constitutively active mutants. (**A**) Ribbon representation of the Ca^2+^-bound mouse TMEM16A channel viewed from within the membrane (PDB: 5OYB), subunits are shown in unique colors. Black solid lines, outer- and innermost membrane boundaries, black dashed lines, boundaries of the hydrophobic core; blue spheres, Ca^2+^ ions; violet surface, molecular surface of the pore (generated in HOLE [Smart et al., 1996] with a solvent probe radius of 0.7 Å). (**B**) Close-up of the intracellular vestibule with selected residues displayed as sticks and labelled. Green sphere, Cα of Gly 644. α3 and α4 are removed for clarity. (**C**) Instantaneous I-V relations of the constitutively active mutant G644P from pre-pulses at 80 mV in the presence of the indicated intracellular Ca^2+^ concentrations. Solid lines are fits to Equation 2. Data were normalized to the fitted amplitude factor (*A* in Equation 2) and were subsequently normalized to the current amplitude at 120 mV at zero Ca^2+^ (I/I _+120mV 0Ca_). Data are mean values of normalized I-V plots from 5 to 13 individual patches, errors are s.e.m. (**D**) Relative energy profiles (at 0 mV) of the ion conduction path at the indicated intracellular Ca^2+^ concentrations (colors as in C). Barriers are visualized as a sum of three Gaussians with the peaks and amplitudes indicating their locations and relative barrier heights respectively. (**E**) Relative rate of barrier crossing at the indicated location (σ_h_ and σ_β_) as a function of intracellular Ca^2+^ concentration. Solid lines are fits to the Hill equation. Data are best-fit values and errors are 95% confidence intervals. (**F**) Instantaneous I-V relations of the mutant Q649A from pre-pulses at 100 mV (left) and −60 mV (right) in the presence of the indicated intracellular Ca^2+^ concentrations. Solid lines are fits to Equation 2. Data were normalized as in C and are mean values of normalized I-V plots from five individual patches, errors are s.e.m. (**G**) Relative rate of barrier crossing at the intracellular pore entrance (σ_β_) as a function of both intracellular Ca^2+^ concentration and voltage. Solid lines are fits to the Hill equation.

The calcium-activated chloride channel TMEM16A is part of a large family of membrane proteins that encompasses ion channels and lipid scramblases with a common conserved molecular architecture (Brunner et al., 2016; Caputo et al., 2008; Picollo et al., 2015; Schroeder et al., 2008; Terashima et al., 2013; Whitlock and Hartzell, 2017; Yang et al., 2008). With respect to their fold, the TMEM16 family is related to mechanosensitive channels of the OSCA and TMC families (Ballesteros et al., 2018; Pan et al., 2018; Zhang et al., 2018). TMEM16A is widely expressed and contributes to important physiological processes including the transport of chloride across epithelia and the control of electrical signal transduction in smooth muscle and certain neurons (Huang et al., 2012; Oh and Jung, 2016; Scudieri et al., 2012). Recent investigations have defined the structural basis for ion permeation and gating in TMEM16A and revealed features that distinguish this ion channel from other homologues working as lipid scramblases (Brunner et al., 2014; Dang et al., 2017; Paulino et al., 2017a; Paulino et al., 2017b). TMEM16A harbors two ion conduction pores, each contained within a single subunit of a homodimeric protein (Figure 1A). Both pores function independently and are activated by the binding of two Ca^2+^ ions to a site embedded within the membrane-inserted part of each subunit close to the ion conduction path (Jeng et al., 2016; Lim et al., 2016). In the open conformation, anions access the narrow neck of an hourglass-shaped pore via water-filled vestibules from the extra- and intracellular sides and permeate through the constricted part presumably after shedding most of their coordinating water molecules (Betto et al., 2014; Dang et al., 2017; Ni et al., 2014; Paulino et al., 2017a; Qu and Hartzell, 2000). During this process, the energetic penalty for dehydration is surmounted by positive charges placed on both sides of the neck (Paulino et al., 2017b). In the apo conformation, the Ca^2+^ binding site is accessible from the cytoplasm (Paulino et al., 2017a). The binding of Ca^2+^ favors conformational changes in the pore-lining helix α6, which provides polar and acidic sidechains that coordinate the bound cations and thus directly couples ligand binding to pore opening (Paulino et al., 2017a; Peters et al., 2018). The subsequent closure of the aqueous access pathway to the ligand binding site buries the bound calcium ions within the transmembrane electric field, which mechanistically accounts for the observed voltage dependence of activation by modulating the binding affinity of Ca^2+^ (Arreola et al., 1996; Brunner et al., 2014; Xiao et al., 2011). The movement of the helix relays a conformational change towards the neck to release a steric barrier, which acts as a gate in the closed conformation of the channel (Paulino et al., 2017a). Additionally, the bound Ca^2+^ ions change the charge distribution at the wide intracellular vestibule thereby removing a second, electrostatic barrier that specifically hinders the access of anions in the ligand-free state (Paulino et al., 2017a). Here we have used electrophysiology and Poisson-Boltzmann calculations to characterize the electrostatic control of anion permeation by the bound Ca^2+^ ions. Our results reveal a strong and favorable Coulombic interaction of permeant anions with the positively charged Ca^2+^ at the intracellular pore entry. These long-range interactions underlie an electrostatic gating mechanism that acts through space and that is synergistic with the opening of a steric gate, allowing an otherwise sub-conductive pore to conduct with full capacity.

**Figure 1—figure supplement 1.**
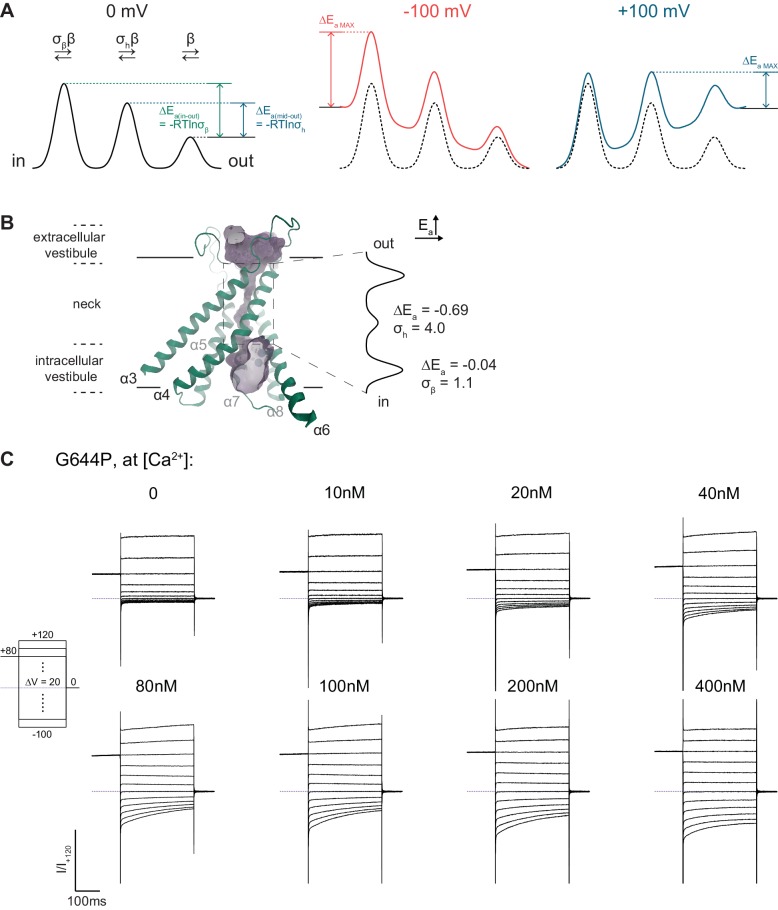
Permeation model and G644P currents. (**A**) Energy profile indicating the relationship between the relative rate constants (σ_h_ and σ_β_) and the energy difference between the middle or inner barrier and the outer barrier at 0 mV (ΔE_a (mid-out)_ and ΔE_a (in-out)_, left). The effect of a constant electric field across the channel is shown (center, right). (**B**) Relationship between the pore and the energy profile of WT. The molecular surface of the pore is shown in violet. Blue spheres, calcium ions. Selected helices are shown and are labelled. View is from within the membrane. (**C**) Rundown-corrected and normalized current responses of G644P used to construct the I-V relations shown in Figure 1C.

**Figure 1—figure supplement 2.**
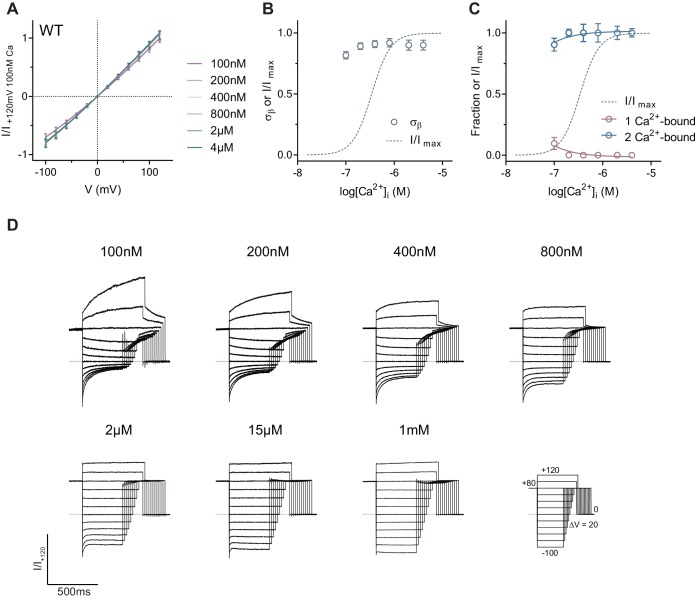
Relative contribution of open states with various Ca^2+^occupancy to the activation of WT. (**A**) Instantaneous I-V relations from pre-pulses at 80 mV in the presence of the indicated intracellular Ca^2+^ concentrations for WT. Solid lines are fits to Equation 2. Data were processed as in Figure 1C and are mean values of normalized I-V plots from 7 to 8 individual patches, errors are s.e.m. (**B**) Relative rate of barrier crossing at the intracellular pore entrance (σ_β_) of WT as a function of intracellular Ca^2+^ concentration. Dashed lines are the concentration-response relation of WT at 80 mV. Data are best-fit values and errors are 95% confidence intervals. (**C**) Relative contribution of open states with various Ca^2+^ occupancy during activation. Data are best-fit values obtained from fitting the data in A to Equation 7, errors are 95% confidence intervals. Solid lines describe the trend and have no theoretical meaning. (**D**) Rundown-corrected and normalized current responses of WT used to construct the I-V relations shown in A. .

**Figure 1—figure supplement 3.**
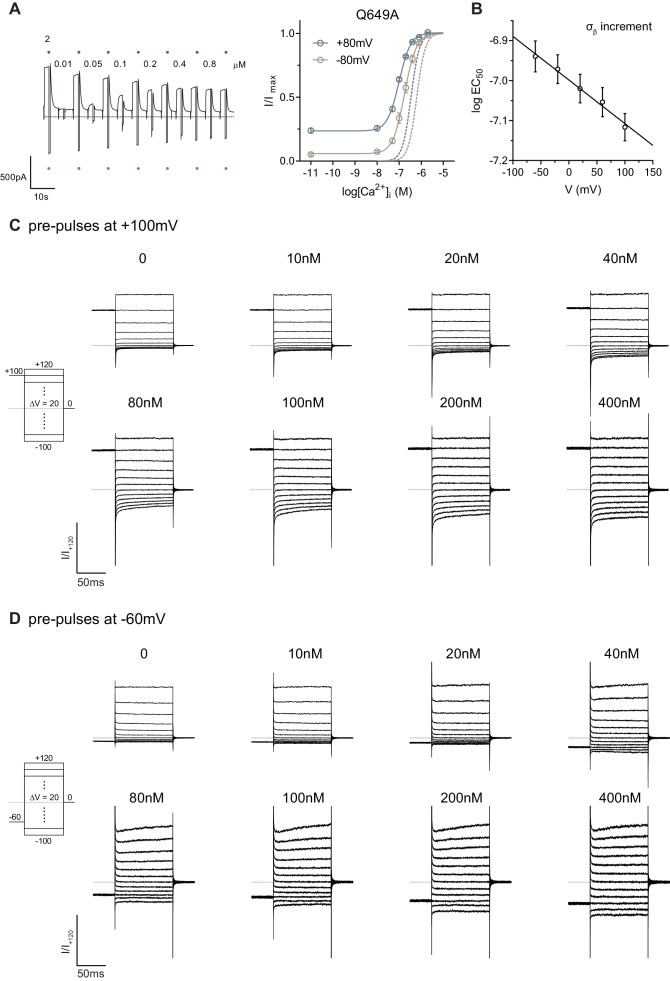
Characterization of the mutant Q649A. (**A**) Representative current trace (left) and concentration-response relations (right) of Q649A recorded at +/-80 mV using a rundown-correction protocol. Solid lines are fits to the Hill equation. Dashed lines are the relations of WT. Data are mean values of normalized concentration-response relations from 6 individual patches, errors are s.e.m. Blue and violet dots on the current trace indicate the reference pulses used for rundown correction. (**B**) EC_50_ for σ_β_ increment (Figure 1G) as a function of voltage. Solid line is a fit to Equation 1 . Data are best-fit values and errors are 95 % confidence intervals. The electrical distance ($f_{V}$) was estimated to be 0.032 ± 0.007. C-D. Rundown-corrected and normalized current responses of Q649A with pre-pulses at, (**C**), 100 mV and, (**D**), -60 mV.

## Results

### Ca^2+^ binding to the transmembrane site alleviates energy barriers for anion conduction

We have previously used a simple rate model to characterize ion conduction in TMEM16A (Läuger, 1973; Paulino et al., 2017b). In this model, a central small energy barrier originating from the diffusion of an anion across the narrow neck is sandwiched between two larger barriers resulting from the desolvation of the anion upon entering the constricted part of the channel from the aqueous vestibules located on either side of the pore (Figure 1A, Figure 1—figure supplement 1A,B). The diffusion path does not contain deep energy wells and the model does not account for saturation of the pore, which is generally consistent with the high K_M_ for chloride conduction. This model allowed us to phenomenologically interpret the effect of mutations of positively charged residues on current-voltage (I-V) relationships. Whereas single mutations in the wide vestibules do not exert a recognizable effect on conduction, mutations at the border of the neck region result in a pronounced rectification of currents whose shape depends on the location of the altered residues (Paulino et al., 2017b). The rectification is a consequence of both pore occupancy and the rate of barrier crossing at the applied potential. Here we use a similar analysis to investigate the influence of the bound calcium ions on anion conduction by characterizing the properties of the mutant G644P. In this mutant, the replacement of a flexible glycine at a hinge in α6 (Gly 644, Figure 1B) with a rigid proline increases the potency of Ca^2+^ and concomitantly results in constitutive activity (Paulino et al., 2017a). The basal current of this mutant is highly outwardly rectifying but it progressively loses its rectification at increasing Ca^2+^ concentrations until it becomes pseudo-linear in saturating conditions (Figure 1C, Figure 1—figure supplement 1C). This differs from the instantaneous currents of WT, which are linear in the entire Ca^2+^ concentration range (Figure 1—figure supplement 2). When analyzed with our described minimal model of ion permeation, this effect seems to originate from the alleviation of local energy barriers for anion conduction at the intracellular entrance and the center of the pore as a function of Ca^2+^ concentration (Figure 1D). This is consistent with a change in long-range interactions affecting permeant anions entering and traversing the narrow neck region upon binding of the positively charged Ca^2+^. The lowering of the energy barriers can be described by a binding isotherm with EC_50_ values of 60 nM and 170 nM, and Hill coefficients of 2.1 and 1.7 at 80 mV for the inner and the central barriers respectively (Figure 1E). As the rate constants are not strongly coupled, the similarity of their respective EC_50_’s and their consistency with data from steady-state current responses provide independent evidence for a saturable effect associated with the binding of Ca^2+^ to the transmembrane site identified in the structure.

Besides G644P, we have characterized the mutant Q649A that also causes constitutive activity. Located about one helix turn below Gly 644, the residue is not directly involved in Ca^2+^ coordination (Figure 1B). Like G644P, this mutant displays a left-shifted EC_50_ and basal current that is equally outwardly rectifying (Figure 1—figure supplement 3A). Since the current magnitude of this mutant is larger and the kinetics of irreversible rundown is slower compared to G644P, we were able to examine the influence of the membrane potential on Ca^2+^ binding by recording instantaneous currents with pre-pulses at different voltages on the same patch (Figure 1F and Figure 1—figure supplement 3B—D). At the membrane potentials tested, we observed a similar effect of Ca^2+^ on the energetics of anion conduction as in G644P (Figure 1C–F) and as with steady-state concentration-responses, the potency of Ca^2+^ is increased upon depolarization (Figure 1F–G, Figure 1—figure supplement 3B–D). Therefore, these results provide further evidence that Ca^2+^ alleviates local energy barriers for anion conduction at the intracellular entrance and the middle of the pore by binding to the transmembrane site.

### The effect of the competitive antagonist Mg^2+^, and the trivalent cation Gd^3+^ on conduction

**Figure 2.**
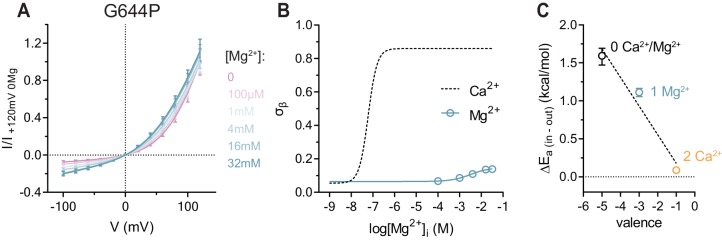
Conduction properties of the constitutively active mutant G644P in the presence of intracellular Mg^2+^. (**A**) Instantaneous I-V relations of the constitutively active mutant G644P from pre-pulses at 80 mV in the presence of the indicated intracellular Mg^2+^ concentrations. Solid lines are fits to Equation 2. Data were normalized to the fitted amplitude factor (*A* in Equation 2) and were subsequently normalized to the current amplitude at 120 mV at zero Mg^2+^ (I/I _+120mV 0Mg_). Data are mean values of normalized I-V plots from 9-11 individual patches, errors are s.e.m. (**B**) Relative rate of barrier crossing at the intracellular pore entrance (σ_β_) as a function of intracellular Mg^2+^ concentration. Solid line is a fit to the Hill equation for one binding site. The relation with Ca^2+^ is shown as dashed line for comparison. Data are best-fit values and errors are 95% confidence intervals. (**C**) Experimental relationship between the assumed net charge of the Ca^2+^ binding site (valence) and anion conduction energetics (ΔE_a (in-out)_). Data are transforms of σ_β_, using Equation 3, at zero and saturating Mg^2+^ and Ca^2+^ concentrations. Dashed Line is a fit to Equation 4. The relative permittivity ($\varepsilon_{r}$) for occupancy by divalent cations was estimated to be 64.8 ± 35.1.

To confirm that the charge of Ca^2+^, rather than its ability to promote gating-associated conformational changes, accounts for the observed effect, we characterized the I-V relations of G644P in the presence of the divalent cation Mg^2+^ (Figure 2). Although Mg^2+^ is incapable of activating WT even at high concentrations (Figure 2—figure supplement 1A), it acts as a low affinity competitive antagonist that lowers the potency of Ca^2+^, indicating that it might occupy the Ca^2+^ binding site (Ni et al., 2014). This is confirmed in the mutant G644P, for which we observed a concentration-dependent reduction of the outward rectification in the presence of Mg^2+^ (Figure 2A). Mg^2+^ thus appears to ‘activate’ G644P, by primarily increasing its conductance but not its open probability (Figure 2—figure supplement 1B and C). Although we cannot exclude a non-specific effect of Mg^2+^ due to the screening of surface charges, this is unlikely, as alanine mutations of positive charges in the wide vestibule have previously been shown to exert little effect on conduction (Paulino et al., 2017b). Interestingly, although Mg^2+^ exerts a qualitatively similar effect on G644P as Ca^2+^, the current remains rectifying even at saturating concentrations (Figure 2B). Moreover, in contrast to Ca^2+^, the activation by Mg^2+^ proceeds with a Hill coefficient close to unity and a maximum conduction rate at the intracellular entrance (σ_β_) that saturates at an intermediate value, suggesting that the binding site is likely occupied by a single Mg^2+^ ion (Figure 2B and C). Taken together, our data show three discrete levels of conductance that correspond to the possible occupancies of the binding site with zero, one and two divalent cations bound (Figure 2C), consistent with a scenario where sequential Ca^2+^ binding lowers the energy barriers for anion conduction in a stepwise manner, as predicted by a simple Coulombic interaction. The model also describes the effect upon addition of the trivalent cation Gd^3+^, which strongly increases the conductance at saturating concentrations even beyond the level induced by Ca^2+^. This is likely a consequence of the increased positive charge density in the binding site and is manifested in the observed inward rectification of currents (Figure 2—figure supplement 2).

**Figure 2—figure supplement 1.**
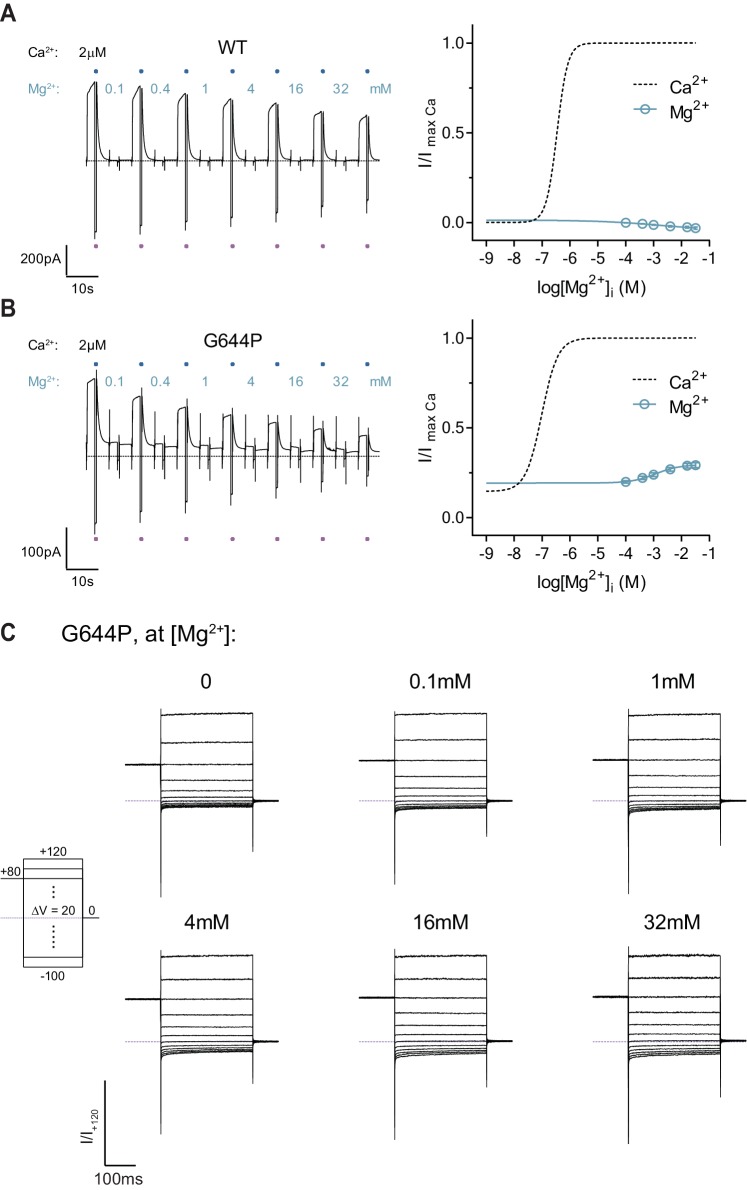
Mg^2+^concentration-response relations of WT and G644P. A-B, Representative current traces (left) and concentration-response relations (right) of WT (**A**) and G644P (**B**) recorded at ±80 mV using a rundown-correction protocol. Solid lines are fits to the Hill equation for one binding site. Dashed lines are the relations with Ca^2+^ shown for comparison. Data are mean values of normalized concentration-response relations from 5 and 7 individual patches respectively, errors are s.e.m. Blue and violet dots on the current traces indicate the reference pulses used for rundown correction. (**C**) Rundown-corrected and normalized current responses of G644P at the indicated Mg^2+^ concentrations used to construct the I-V relations shown in Figure 2A.

**Figure 2—figure supplement 2.**
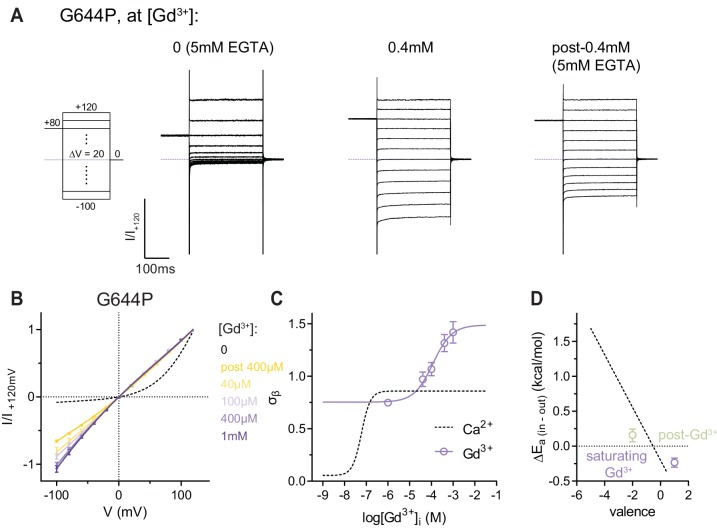
Conduction properties of G644P in the presence of intracellular Gd^3+^. (**A**) Rundown-corrected and normalized current responses of G644P at the indicated Gd^3+^ concentrations. A slowly dissociating occupancy persists upon a jump to 5 mM EGTA post-0.4 mM Gd^3+^. (**B**) Instantaneous I-V relations of G644P from pre-pulses at 80 mV in the presence of the indicated intracellular Gd^3+^ concentrations. Solid lines are fits to Equation 2. Data were normalized to the respective current amplitudes at 120 mV (I/I_+120_). Data are mean values of normalized I-V plots from 3 to 9 individual patches, errors are s.e.m. (**C**) Relative rate of barrier crossing at the intracellular pore entrance (σ_β_) as a function of intracellular Gd^3+^ concentration. Solid line is a fit to the Hill equation for one binding site. The relation with Ca^2+^ is shown as dashed line for comparison. Data are best-fit values and errors are 95% confidence intervals. (**D**) Anion conduction energetics (ΔE_a (in-out)_) at saturating Gd^3+^ concentrations and after a jump to 5 mM EGTA post-0.4mM Gd^3+^. Data are transforms of σ_β_, using Equation 3, at the two plateaus determined in C. Dashed line indicates the relation between divalent occupancy and anion conduction energetics determined with Mg^2+^ and Ca^2+^ from Figure 2C. The extra valence due to Gd^3+^ binding were assigned as 3 and 6, which might not necessarily be the case as a mixed occupancy by Gd^3+^ and other trace di/trivalent cations cannot be ruled out under the recording conditions.

### Ca^2+^ increases anion conductance by an electrostatic mechanism

**Figure 3.**
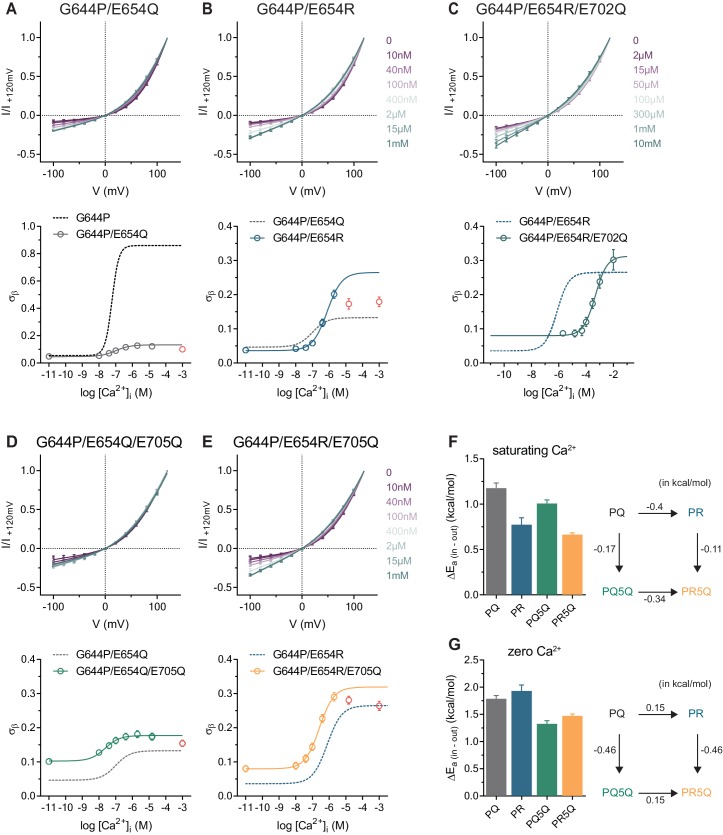
Conduction properties of the constitutively active mutant G644P with additional mutations at the Ca^2+^binding site. A-E, Top, instantaneous I-V relations from pre-pulses at 80 mV in the presence of the indicated intracellular Ca^2+^ concentrations for mutants G644P/E654Q (**A**), G644P/E654R (**B**), G644P/E654R/E702Q (**C**), G644P/E654Q/E705Q (**D**) and G644P/E654R/E705Q (**E**). Solid lines are fits to Equation 2. Data were normalized to the current amplitude at +120 mV of each curve and are mean values of normalized I-V plots from 6, 7, 5–10, 5 and 7 individual patches respectively, errors are s.e.m. Bottom, relative rate of barrier crossing at the intracellular pore entrance (σ_β_) as a function of intracellular Ca^2+^ concentration for mutants G644P/E654Q (**A**), G644P/E654R (**B**) G644P/E654R/E702Q (**C**), G644P/E654Q/E705Q (**D**) and G644P/E654R/E705Q (**E**). Solid lines are fits to the Hill equation for one binding site. Data in red were omitted from the fit (see Materials and methods). Dashed lines are the relations of the indicated mutant shown for comparison. Data are best-fit values and errors are 95% confidence intervals. F-G, Left, relative activation energies at the intracellular pore entrance (ΔE_a (in-out)_) for the indicated mutants at saturating Ca^2+^ concentrations (**F**) and zero Ca^2+^ (**G**). Data are transforms of σ_β_, using Equation 3, at saturating and zero Ca^2+^ concentrations obtained from the fits shown in A-E. Right, energetic changes from the parent mutant G644P/E654Q (PQ) in a double-mutant cycle for the indicated mutants at saturating Ca^2+^ concentrations (**F**) and zero Ca^2+^ (**G**).

Our results suggest that Ca^2+^ and other di- and trivalent cations gate anion conduction in both constitutively active mutants by an electrostatic mechanism that involves neutralization of the negative charges in the vacant binding site. To gain further insight into this process, we investigated the effect of mutations of residues at the Ca^2+^ binding site introduced in the G644P background and initially focused on E654Q located on α6. Whereas on a WT background, E654Q exhibits the most severe phenotype among binding site mutants and does not show any activity even at high Ca^2+^ concentrations (Brunner et al., 2014; Lim et al., 2016; Tien et al., 2014), we observed basal and outwardly-rectifying currents in the mutant G644P/E654Q (PQ) whose steady-state current increases in response to Ca^2+^ addition (Figure 3—figure supplement 1A). We examined the instantaneous I-V relations at increasing Ca^2+^ concentrations and found a moderate concentration-dependent reduction of rectification that eventually saturates (Figure 3A). The much lower conduction rate of the PQ mutant compared to G644P at saturating Ca^2+^ concentrations and a Hill coefficient close to one both suggest that in this mutant the binding of a single calcium ion to the transmembrane site promotes anion permeation, although less efficiently than the binding of two calcium ions in G644P, similar to the effect of Mg^2+^ (Figures 2 and 3A).

**Figure 3—figure supplement 1.**
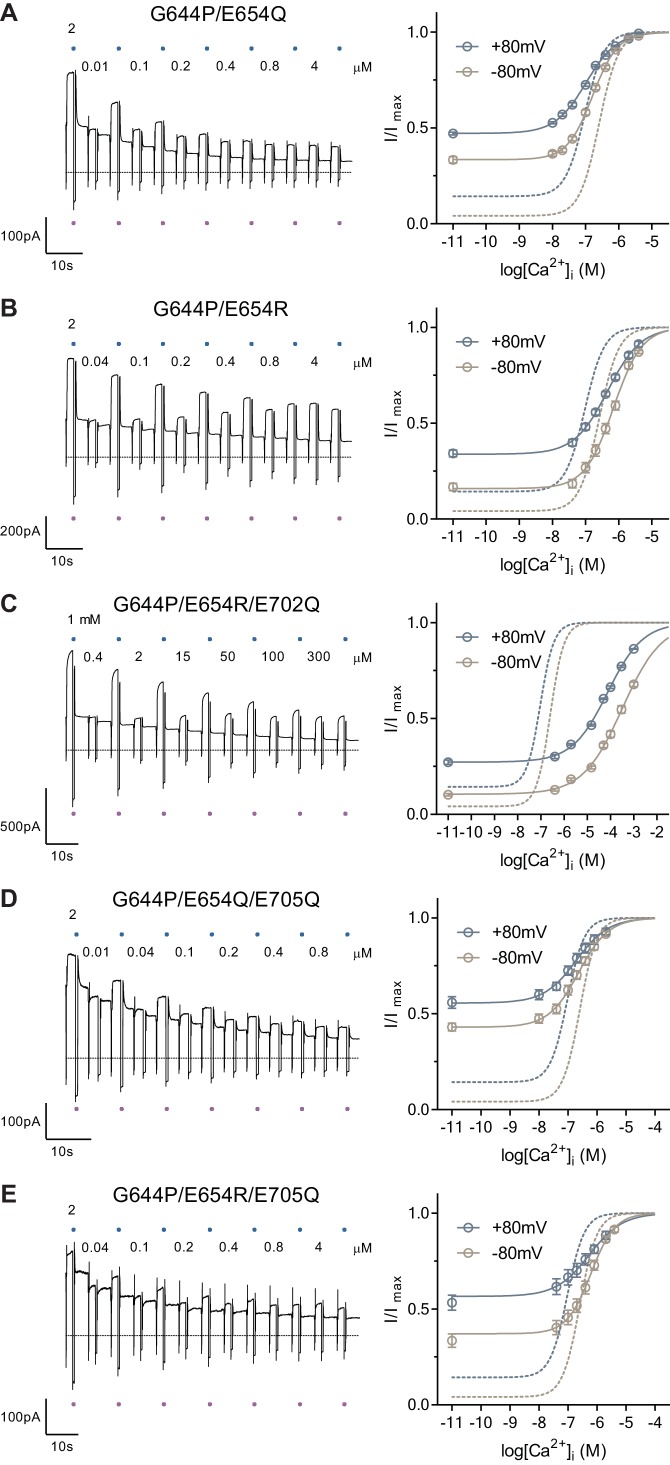
Concertation-response relations of mutants. Representative current traces (left) and concentration-response relations (right) of mutants G644P/E654Q (**A**), G644P/E654R (**B**) G644P/E654R/E702Q (**C**), G644P/E654Q/E705Q (**D**) and G644P/E654R/E705Q (**E**) recorded at ±80 mV using a rundown-correction protocol. Solid lines are fits to the Hill equation. Dashed lines are the relations of G644P shown for comparison. Data are mean values of normalized concentration-response relations from 4 to 12, 8 – 11, 5 – 7, 6 – 7 and 10 individual patches respectively, errors are s.e.m. Blue and violet dots on the current traces indicate the reference pulses used for rundown correction.

We extended our investigation on Glu 654 and mutated this residue to an arginine (PR) to add a further positive charge to the binding site. Similar to PQ, this mutant likely binds only one calcium ion as the Hill coefficient is close to unity and the I-V relation retains its strong rectification even at saturating Ca^2+^ concentrations (Figure 3B, Figure 3—figure supplement 1B). However, consistent with the hypothesis that a more positive potential at the binding site lowers the energy barrier for anion permeation, the rate of chloride conduction at the intracellular entrance at saturating Ca^2+^ concentrations is significantly higher than that observed for PQ (Figure 3B). Next, we removed an additional negative charge in the binding site of the PR mutant by exchanging Glu 702 with Gln (PR2Q), which, similar to the equivalent mutation on the WT background (Brunner et al., 2014), reduces the potency of Ca^2+^ (Figure 3C, Figure 3—figure supplement 1C). As expected, the rate of chloride conduction at the intracellular pore entrance in the triple mutant PR2Q is increased compared to the double mutant PR both in the absence of Ca^2+^ and at saturating Ca^2+^ concentrations (Figure 3C). Together these observations consolidate the notion of the dependence of anion conduction on the electrostatic potential around the binding site.

For electrostatic interactions, we expect the contribution of individual charges in the binding site to conduction to be additive. This hypothesis was tested by analyzing the effect of the removal of a negative charge in the binding site mutant E705Q in the PQ (PQ5Q) and PR (PR5Q) backgrounds. At maximum Ca^2+^ occupancy, E705Q increases the rate of anion conduction to the same extent in both PQ5Q and PR5Q irrespectively of its background (Figure 3D,E), which emphasizes the orthogonal contributions of individual mutations (Figure 3F). The same mutations also exhibit a similar degree of additivity in the Ca^2+^-free state (Figure 3G), further supporting an electrostatic mechanism for the functional interaction between the Ca^2+^ binding site and the anion conduction path.

### Electrostatic interactions are evident in calculations

**Figure 4.**
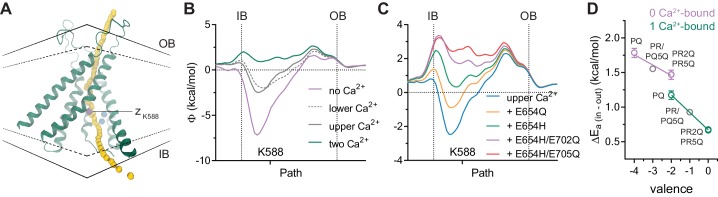
Electrostatic profiles. (**A**) System in which the electrostatic profile of the pore was calculated. Selected helices are shown and view is from within the membrane on one of the two pores in the dimeric protein. Black solid lines, outer- and innermost membrane boundaries (OB and IB, respectively); black dashed lines, boundaries of the hydrophobic core; blue spheres, Ca^2+^ ions; yellow spheres, points at which the electrostatic potential (Φ) was plotted. Magenta sphere corresponds to the z position of the nitrogen atom of Lys 588. B-C. Electrostatic potential along the pore in the Ca^2+^-bound structure containing the indicated number of Ca^2+^ ions (**B**) and carrying the indicated *in silico* mutations with only the upper Ca^2+^ ion bound (**C**). Vertical dashed lines indicate the membrane boundaries. The position of Lys 588 at the intracellular pore entrance is indicated. (**D**) Experimental relationship between the assumed net charge of the Ca^2+^ binding site (valence) and anion conduction energetics (ΔE_a (in-out)_). Data are transforms of σ_β_, using Equation 3, at saturating and zero Ca^2+^ concentrations for all the mutants on the G644P background (Figure 2). Lines are fits to Equation 4. The relative permittivity ($\varepsilon_{r}$) for the 0 Ca^2+^-bound and 1 Ca^2+^-bound states were estimated to be 162.7 ± 127.2 and 96.7 ± 6.3 respectively.

To further strengthen our proposal of an electrostatic interaction between the bound Ca^2+^ and permeating anions, we investigated the effect of the binding site mutations and the altered Ca^2+^ occupancy on the electrostatic profile of the pore using Poisson-Boltzmann calculations on the Ca^2+^-bound mouse TMEM16A structure (Figure 4A). Consistent with the quantal effect of Ca^2+^ on anion conduction, removal of a single calcium ion from the binding site partially decreases the positive potential observed in the fully bound structure (Figure 4B). We also found that stepwise removal of charges in the binding site exerts progressively larger effects according to the number of charges neutralized (Figure 4C), as observed in our experiments. Although we cannot exclude the possibility that the detailed geometric arrangement of the Ca^2+^ binding site and nearby helices might be influenced by the introduced mutations, local changes in backbone and sidechain conformations are unlikely to drastically affect the calculated electrostatic potential given the long-range nature of Coulombic interactions. Because several mutants described above seem to bind only one calcium ion (Figure 3 and Figure 3—figure supplement 1), it is possible to estimate from our experimental data the effective dielectric constant of the intracellular vestibule that propagates the electric field originating from the binding site to the pore. When the above mutants are analyzed collectively, we observed an inverse and linear relationship between the energy barrier for chloride conduction at the intracellular pore entrance and the valence of the binding site (Figure 4D). Assuming a Coulombic potential and using the distance measured from the Ca^2+^-bound structure, we estimated an effective dielectric constant of around 160 in the vacant and 90 for the single Ca^2+^-bound state. Although potentially inaccurate and physically unreasonable due to experimental limitations, these values indicate bulk solution-like aqueous properties that are consistent with the large water-filled intracellular vestibule observed in the Ca^2+^-bound structure (Figure 4D). This also suggests that the vestibule is functionally shielded from the membrane.

### Bidirectional electrostatic interaction between the binding site and the intracellular pore entrance

**Figure 5.**
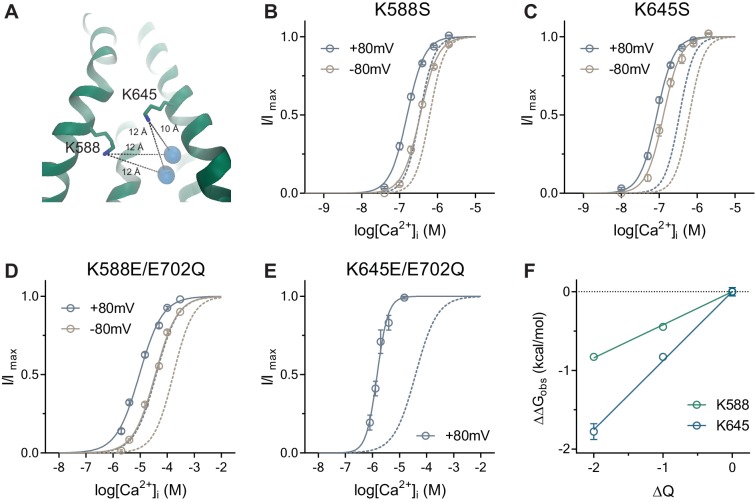
Activation properties of pore mutants. (**A**) Location of the pore residues Lys 588 and 645 relative to the Ca^2+^ binding site (distances in Å). B-E. Concentration-response relations of K588S (**B**), K645S (**C**), K588E/E702Q (**D**) and K645E/E702Q (**E**) recorded at +/-80 mV using a rundown-correction protocol. Solid lines are fits to the Hill equation. Dashed lines are the relations of WT (**B–C**) and E702Q (**D–E**). Data are mean values of normalized concentration-response relations from 8-11, 7-8, 10 and 5-7 individual patches respectively, errors are s.e.m. (**F**) Relationship between the electrostatic potential of the intracellular pore entrance (ΔQ) and Ca^2+^ binding energetics (ΔΔG_obs_) for the indicated residues. Data are transforms of EC_50 mutant_/EC_50 background_ at +80 mV using Equation 6. Solid lines are fits to Equation 5. The relative permittivity ($\varepsilon_{r}$) for Lys 588 and 645 were estimated to be 131.9 ± 12.3 and 71.2 ± 6.1 respectively.

In previous experiments, we have demonstrated that the bound Ca^2+^ ions influence ion permeation by affecting the electrostatics at the narrow neck region via long-range Coulombic interactions. Reciprocally, changes in the charge distribution at the neck should affect Ca^2+^ binding. To test this hypothesis, we have investigated the effect of mutations of two residues, Lys 588 and Lys 645 (Figure 5A) located at the boundary between the intracellular vestibule and the neck, which lower the energy barrier for chloride conduction by contributing to the positive electrostatics of the pore (Paulino et al., 2017b). As expected, we found an increase in the potency of Ca^2+^ in neutralizing mutations of the respective residues to serine (Figure 5B,C), which is further enhanced upon the reversal of the charge by mutations to glutamate (Figure 5D,E). Assuming that only the binding affinity of Ca^2+^ is affected, we observed a linear relationship between the change in binding energy and the valence at the intracellular pore entrance (Figure 5F), with a stronger sensitivity for Lys 645, which is in closer proximity to the Ca^2+^ binding site (Figure 5A) and thus might experience less solvent screening. In summary, the described effects further confirm the long-range interactions between the Ca^2+^ binding site and the narrow neck region, which underlie the electrostatic control of ion permeation.

**Figure 5—figure supplement 1.**
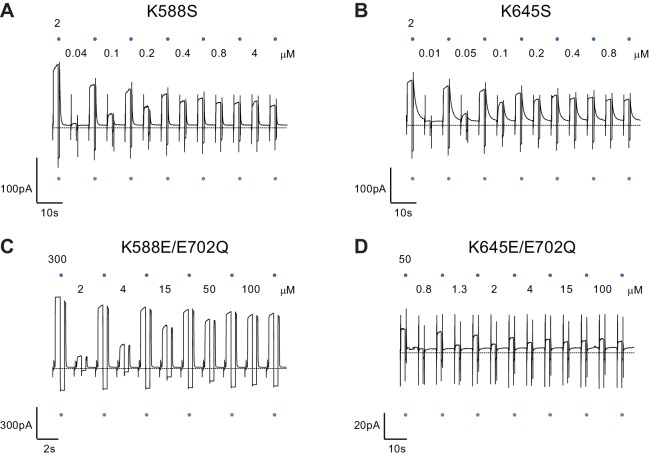
Current traces of mutants at the neck region. Representative current traces of mutants K588S (**A**), K645S (**B**), K588E/E702Q (**C**) and K645E/E702Q (**D**). Blue and violet dots indicate the reference pulses used for rundown correction.

## Discussion

**Figure 6.**
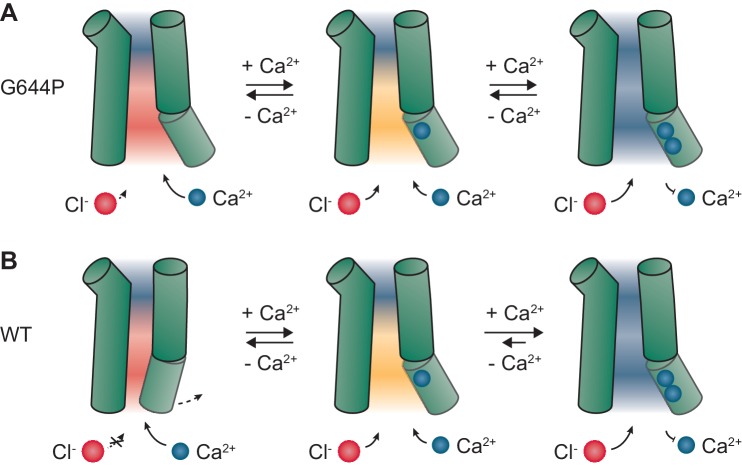
Mechanism. Schematic depiction of gating in G644P (**A**) and WT (**B**). In the apo state of the mutant G644P, the negative electrostatic potential strongly favors the access of Ca^2+^ over Cl^-^. In contrast, the positive electrostatic potential in the doubly occupied state strongly favors the access of Cl^-^. A similar mechanism is expected to occur in WT, which requires Ca^2+^ for the activation of α6 and the opening of a steric gate located above the intracellular vestibule. When activated, the major conducting state in WT is the open state with two Ca^2+^ ions bound. Green cylinders, selected helices delimiting the pore; blue spheres, Ca^2+^ ions; red spheres, Cl^-^ ions. Red, orange and blue backgrounds depict negative, mildly negative and positive electrostatic potential in the pore.

Our study demonstrates the strong electrostatic influence of bound Ca^2+^ ions on anion conduction in the Ca^2+^-activated Cl^-^ channel TMEM16A. A direct effect of the ligand on the permeation properties of the open state is unique among ligand-gated ion channels and it is a consequence of the high positive charge density of Ca^2+^ and the location of its binding site in the immediate vicinity of the permeation path. The described effect is synergistic with a conformational change upon Ca^2+^ binding that opens a steric gate in the narrow part of the pore (Paulino et al., 2017a). In the absence of Ca^2+^, even in a sterically released conformation, which in this study was stabilized by mutations, an electrostatic gate imposed by the vacant binding site would impede the access of chloride ions to the intracellular vestibule (Figure 6). The binding of Ca^2+^ releases this second gate by neutralizing the negative charges, enabling the open channel to conduct with full capacity (Figure 6). Our findings thus provide novel mechanistic insight into the regulation of anion permeation in Ca^2+^-activated Cl^-^ channels of the TMEM16 family and demonstrate that these channels are dually gated by both steric and electrostatic mechanisms. Moreover, they also provide a conceivable explanation for how the oppositely charged substrates, in this case permeating anions and Ca^2+^ ions, access their respective site of action via the same intracellular vestibule (Figure 6). The observation that the conductance of the open channel can be electrostatically regulated by Ca^2+^ in a stepwise manner also offers a unique opportunity to examine the relative contribution of open states with partial Ca^2+^ occupancy during activation. The currents in such partially occupied states would be rectifying and thus be readily identifiable in the current-voltage relationships. In WT, the instantaneous I-V plots are all approximately linear and their shape is only mildly sensitive to Ca^2+^ in the entire activation range (Figure 1—figure supplement 2). Since these plots reflect the weighted sum of the I-V relations of the open states with different Ca^2+^ occupancy, our results indicate that, during activation, the major conducting state in WT is the open state with two Ca^2+^ ions bound, whereas the contribution of open states with submaximal Ca^2+^ occupancy is minimal (Figure 1—figure supplement 2 and Figure 6—figure supplement 1A,B). A similar but less pronounced distribution of states is also evident in the constitutively active mutant G644P (Figure 6—figure supplement 1C), where the apo and the singly occupied channel make a larger contribution to conduction at low Ca^2+^ concentrations. These observations thus provide direct evidence for the high cooperativity of TMEM16A activation.

**Figure 6—figure supplement 1.**
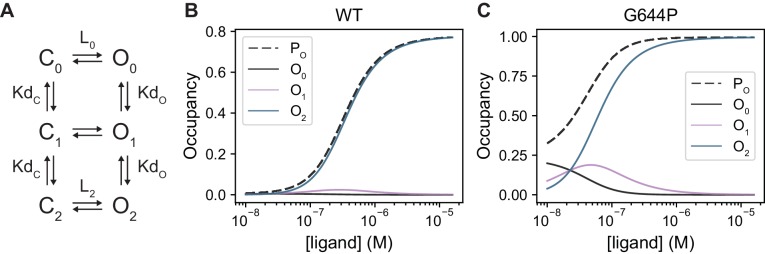
Contribution of apo-, singly- and doubly occupied states in WT and G644P in a model of activation. (**A**) Minimal model for TMEM16A activation. C and O indicate closed and open states, respectively; the subscript indicates the number of bound Ca^2+^ ions.Kd, equilibrium dissociation constant;L, forward equilibrium constant of gating transitions. Microscopic reversibility requires that${Kd}_{O}={Kd}_{C}\sqrt{\frac{L_{0}}{L_{2}}}$. B-C. Occupancy of open states in relation to the open probability (P_O_) as a function of Ca^2+^ concentration. $L_{0}$= 5 x 10^−3^, $L_{2}$= 3.5 and ${Kd}_{C}$ = 6 x 10^−7^ M for WT (**B**) and $L_{0}$ = 0.3, $L_{2}$= 210 and ${Kd}_{C}$ = 6 x 10^−7^ M for G644P (**C**). The gating constants were set 60 times larger in (**C**) while the affinity was kept unchanged. Parameters were chosen to account for the qualitative behaviour of the constructs and may not be quantitatively exact. Nonetheless, this model is consistent with the degree of cooperativity observed in steady-state responses and our experimental estimates on the relative contribution of open states with various occupancy in WT.

Aside from their impact on membrane biophysics, our findings are particularly relevant for the development of allosteric activators of TMEM16A that may be beneficial for the treatment of obstructive airway diseases such as Cystic Fibrosis (Li et al., 2017; Mall and Galietta, 2015). Although we anticipate that small molecules have the potential to increase the low open probability of the channel at low Ca^2+^ concentration, this might not suffice to promote robust Cl^-^ efflux required to compensate for the loss of the chloride channel CFTR unless at the same time the electrostatic barrier at the intracellular pore entry is released, which would be the case for compounds with a positive net-charge or ones that at the same time increase the potency of Ca^2+^.

## Materials and methods

**Key resources table:** 

Reagent type (species) or resource	Designation	Source or reference	Identifiers	Additional information
Gene (Mus musculus)	TMEM16A or Ano1 splice variant *ac*	DOI: 10.1038/ nature13984	UniProt identifier: Q8BHY3-1	
Cell line (Homo sapiens)	HEK293T	ATCC	ATCC CRL-1573	Obtained directly from ATCC; tested negative for myc oplasma contamination
Transfected construct (Mus musculus)	G644P	this paper		generated using a modified QuikChange protocol as described in Methods
Transfected construct (Mus musculus)	Q649A	this paper		as G644P
Transfected construct (Mus musculus)	G644P/E654Q	this paper		as G644P
Transfected construct (Mus musculus)	G644P/E654R	this paper		as G644P
Transfected construct (Mus musculus)	G644P/E6 54R/E702Q	this paper		as G644P
Transfected construct (Mus musculus)	G644P/E6 54Q/E705Q	this paper		as G644P
Transfected construct (Mus musculus)	G644P/E654R/E705Q	this paper		as G644P
Transfected construct (Mus musculus)	K588S	this paper		as G644P
Transfected construct (Mus musculus)	K645S	this paper		as G644P
Transfected construct (Mus musculus)	K588E/E702Q	this paper		as G644P
Transfected construct (Mus musculus)	K645E/E702Q	this paper		as G644P
Recombinant DNA reagent	modified pcDNA3.1 vector	Invitrogen, DOI: 10.1085/jgp.201611650		bearing a 5′ untranslated region (UTR) of hVEGF (from pcDNA4/ HisMax; Invitrogen)
Software, algorithm	Clampex 10.6	Molecular devices		
Software, algorithm	Clampfit 10.6	Molecular devices		
Software, algorithm	Prism 5/6	GraphPad		
Software, algorithm	Excel	Microsoft		
Software, algorithm	NumPy	http://www.numpy.org/		
Software, algorithm	CHARMM	https://www. charmm.org/		
Software, algorithm	VMD	https://www.ks.uiuc.edu/Research/vmd/		

### Molecular biology and cell culture

HEK293T cells (ATCC CRL-1573) were maintained in Dulbecco’s modified Eagle’s medium (DMEM; Sigma-Aldrich) supplemented with 10 U/ml penicillin, 0.1 mg/ml streptomycin (Sigma-Aldrich), 2 mM L-glutamine (Sigma-Aldrich), and 10% FBS (Sigma-Aldrich) in a humidified atmosphere containing 5% CO_2_ at 37 °C. HEK293T cells were transfected with 3 μg DNA per 6 cm Petri dish using the calcium phosphate co-precipitation method, and were used within 24–96 hr after transfection. Mutants were generated with a modified QuikChange method (Zheng et al., 2004) using the wild-type mouse TMEM16A(*ac*) as the template. Double and triple mutants were generated sequentially. All constructs were verified by sequencing.

### Electrophysiology

Recordings were performed on inside-out patches excised from cells expressing the construct of interest. Recording pipettes were pulled from borosilicate glass capillaries (O.D. 1.5, I.D. 0.86, Sutter Instrument) and were fire-polished with a microforge (Narishige) before use. Pipette resistance was typically 3–8 MΩ when standard recording solutions were used. Seal resistance was typically 4 GΩ or higher. Voltage-clamp recordings were performed using Axopatch 200B and Digidata 1550 (Molecular devices). Analogue signals were filtered through the in-built 4-pole Bessel filter at 5 kHz and were digitized at 10–20 kHz. Data acquisition was performed using Clampex 10.6 (Molecular devices). Solution exchange was achieved using a double-barreled theta glass pipette mounted on an ultra-high speed piezo-driven stepper (Siskiyou). Liquid junction potential was found to be consistently negligible given the ionic composition of the solutions and was therefore not corrected. All experiments were performed at 20 °C.

Recordings were performed under symmetrical ionic conditions. Stock solution with Ca^2+^-EGTA contained 150 mM NaCl, 5.99 mM Ca(OH)_2_, 5 mM EGTA and 10 mM HEPES at pH 7.40. Stock solution with EGTA contained 150 mM NaCl, 5 mM EGTA and 10 mM HEPES at pH 7.40. Free Ca^2+^ concentrations were adjusted by mixing the stock solutions at the required ratios, which were calculated using the WEBMAXC program (http://web.stanford.edu/~cpatton/webmaxcS.htm). Recording pipettes were filled with stock solution with Ca^2+^-EGTA, which has a free Ca^2+^ concentration of 1 mM.

For Mg^2+^ experiments, stock solution with Mg^2+^-EGTA contained 75 mM MgCl_2_, 23 mM (NMDG)_2_SO_4_, 5 mM EGTA and 10 mM HEPES at pH 7.40. Free Mg^2+^ concentrations were adjusted by mixing the stock solutions with Mg^2+^-EGTA and EGTA at the required ratios. For Gd^3+^ experiments, the final Gd^3+^ concentrations were reconstituted from a 1 M GdCl_3_ stock in a solution containing 150 mM NaCl, 10 mM HEPES at pH 7.40 with no added Ca^2+^ and metal chelators. In all cases, solutions were prepared in ultrapure molecular biology grade water having a resistivity of 15–18 MΩ cm at 25 °C (ELGA or Millipore).

Concentration-response relations were constructed from steady-state current responses recorded using a rundown-correction protocol as described previously (Lim et al., 2016; Paulino et al., 2017a). For instantaneous I-V relations, current responses were measured at the time points (within milliseconds) where the capacitive current at 0 mV, the membrane potential at which the current reverses, has decayed. The sequence of the applied voltage steps was from −100 to +120 mV to minimize the effect of rundown on the smaller inward current (Figure 1—figure supplements 1–3, Figure 2—figure supplement 1). In a typical recording, the magnitude of the steady-state current at +80 mV would have decayed irreversibly by 10–15% by the time the test pulse at +120 mV is applied. To correct for this effect, the I-V plots were normalized to the current amplitude of the pre-pulses (Paulino et al., 2017b) before their normalization (I/I_+120_). When used judiciously, this procedure allows one to recover the true I-V relation and to correct for current fluctuation. Before and after each recording at the Ca^2+^ concentration of interest, we also recorded in zero Ca^2+^ to ensure that the quality of the patch did not deteriorate over time. Background subtraction was performed for constructs that do not display visible basal activity but was not possible for the mutants that do. In all cases, leaky patches were discarded.

A similar procedure was used for experiments with Mg^2+^ and Gd^3+^. In contrast to the divalent cations Ca^2+^ and Mg^2+^, Gd^3+^ binding appears to be biphasic consisting of a reversible phase and an irreversible component that both affect the I-V relation of the instantaneous current (Figure 2—figure supplement 2A). In order to titrate the second reversible site, we first incubated the patch with a submaximal concentration of Gd^3+^ (0.4 mM) to saturate the irreversible binding site. We then recorded at the test concentration of Gd^3+^ to obtain the corresponding instantaneous I-V relations (Figure 2—figure supplement 2B,C).

### Data analysis

Concentration-response data were fitted to the Hill equation

$$\frac{I}{I_{max}}=\frac{1}{1+10^{(log{EC}_{50}-log[{Ca}^{2+}])h}}$$

where $I/I_{max}$ is the normalized current, ${EC}_{50}$ is the concentration at which $I/I_{max}$ equals 0.5 and h is the Hill coefficient. For the case of one binding site, h was set to equal 1. The voltage dependence of ${EC}_{50}$ was fitted to

(1) $$log{EC}_{50}=log{EC}_{50\left(0\right)}-\frac{1}{2.303}\frac{z_{Ca}f_{V}VF}{RT}$$

where $z_{Ca}$ is the valence of Ca^2+^, $f_{V}$ is the electrical distance, V is the membrane potential, R, T and F have their usual meanings, and ${EC}_{50\left(0\right)}$ is the value of ${EC}_{50}$ when V= 0.

I-V data were fitted to a minimal permeation model that accounts for the most fundamental biophysical behavior of mouse TMEM16A as described previously (Läuger, 1973; Paulino et al., 2017b),

(2) $$I=zFAe^{\frac{zFV}{2nRT}}\frac{c_{i}-c_{o}e^{-\frac{zFV}{RT}}}{e^{-zFV\frac{n-1}{nRT}}+\left(\frac{1}{\sigma_{h}}\right)\frac{1-e^{-zFV\frac{n-2}{nRT}}}{e^{\frac{zFV}{nRT}}-1}+\frac{1}{\sigma_{\beta }}}$$

where I is the current, n is the number of barriers, $c_{i}$ and $c_{o}$ are the intracellular and extracellular concentrations of the charge carrier, z is the valence of Cl^-^ and V, R, T and F are defined as above. $A=\beta_{0}v$ is a proportionality factor where $\beta_{0}$ is the value of β when V= 0 and v is a proportionality coefficient that has a dimension of volume. $\sigma_{h}$ and $\sigma_{\beta }$ are respectively the rate of barrier crossing at the middle and the innermost barriers relative to that at the outermost barrier (β),

$$\sigma_{h}=\frac{h_{V}}{\beta_{V}}=\frac{h_{0}e^{\frac{zFV}{2nRT}}}{\beta_{0}e^{\frac{zFV}{2nRT}}}=\frac{h_{0}}{\beta_{0}}$$

$$\sigma_{\beta }=\frac{\delta_{V}}{\beta_{V}}=\frac{\delta_{0}e^{\frac{zFV}{2nRT}}}{\beta_{0}e^{\frac{zFV}{2nRT}}}=\frac{\delta_{0}}{\beta_{0}}$$

where $h_{0}$ is the rate of barrier crossing at the middle barrier and $\delta_{0}$ at the innermost barrier in the absence of voltage. Thus, for a linear voltage drop as is the case in our model, $\sigma_{h}$ and $\sigma_{\beta }$ are intrinsically voltage-independent and represent the relative rates when V= 0. $\sigma_{\beta }$ appears to be voltage-dependent in Figure 1G because $\sigma_{\beta }$ is a function of Ca^2+^ binding whose EC_50_ exhibits voltage dependence.

In this model, current rectification is governed by the two parameters $\sigma_{h}$ and $\sigma_{\beta }$. Values of $\sigma_{h}$ and $\sigma_{\beta }$ that alter the symmetry of the energy profile (as exemplified in Figure 1—figure supplement 1A ) results in asymmetric I-V relations depending on the directionality of symmetry mismatch (Paulino et al., 2017b). We have previously shown that the model describes the behavior of WT best when the number of barriers nequals 3 (Paulino et al., 2017b), which was used as a fixed parameter. This leaves $\sigma_{h}$, $\sigma_{\beta }$ and the amplitude factor A as the only free parameters to be fitted. In this study, we focused on $\sigma_{\beta }$ because its best-fit value is generally better defined and it increases monotonically with the asymmetry of the I-V relation (rectification index (RI), I_-100/+120_), which allows a straightforward comparison with the I-V data.

The Ca^2+^ dependence of $\sigma_{\beta }$ and $\sigma_{h}$ was fitted to

$$\sigma_{i}=\sigma_{i(min)}+\frac{\sigma_{i(max)}-\sigma_{i(min)}}{1+10^{(log{EC}_{50}-log[{Ca}^{2+}])h}}$$

where the subscript i indicates β or h. We observed for mutants G644P/E654Q, G644P/E654R, G644P/E654Q/E705Q and G644P/E654R/E705Q a low affinity decay of $\sigma_{\beta }$ at high Ca^2+^ concentrations (Figure 3A, B, D, E). Since this decay might be related to processes not intrinsic to the transmembrane binding site (Lim et al., 2016), only the high affinity phase was analyzed. The best-fit values of $\sigma_{\beta }$ at zero and saturating Ca^2+^ concentrations were used to calculate $\Delta E_{a\;\left(in-out\right)}$, the difference between the activation energy at the innermost barrier relative to that of the outermost, using

(3) $$\Delta E_{a\;\left(in-out\right)}=-RTln\sigma_{\beta }$$

The relationship between $\Delta E_{a\;\left(in-out\right)}$ and the valence of the binding site ($z_{bs}$) at different discrete Ca^2+^ occupancies (0, 1 or 2) was fitted to the Coulombic potential

(4) $$\Delta E_{a\;\left(in-out\right)}=\frac{-N_{A}z_{bs}q^{2}}{4\pi \varepsilon_{0}\varepsilon_{r}r}+\Delta E_{a\;\left(in-out\right)\;z_{bs}=0}$$

where $N_{A}$ is the Avogadro’s number, q is the electronic charge, $\varepsilon_{0}$ is the permittivity of vacuum, $\varepsilon_{r}$ is the relative permittivity of the medium, and r is the distance between the binding site and the location of the innermost barrier. $z_{bs}$ was considered 0 when the five acidic residues (Glu 654, 702, 705, 734 and Asp 738) in the binding site are neutralized. $\varepsilon_{r}$ was estimated by setting r to the measured distance between the center of the two calcium ions in the binding site and the sidechain oxygen atom of Ser 592 (r= 13.6 Å) in the structure of mouse TMEM16A in the Ca^2+^-bound state.

An analogous expression was used to account for the changes in the energy of Ca^2+^ binding as a function of valence of the intracellular pore entrance,

(5) $$\Delta \Delta G_{obs}=\frac{N_{A}\Delta z_{pore}z_{Ca}q^{2}}{4\pi \varepsilon_{0}\varepsilon_{r}r}+\Delta \Delta G_{obs\;\Delta z_{pore}=0}$$

where $\Delta \Delta G_{obs}$ is the observed change in the free energy of Ca^2+^ binding when only affinity is affected, ${∆z}_{pore}$ is the valence at the intracellular pore entrance and $z_{Ca}$ is the valence of Ca^2+^. ${∆z}_{pore}$ was considered 0 for the WT protein. $\varepsilon_{r}$ was estimated by setting r to the measured distance between the center of the two calcium ions in the binding site and the sidechain nitrogen atoms of Lys 588 or 645 (r= 11.9 Å or 10.6 Å) in the structure of mouse TMEM16A in the Ca^2+^-bound state. $\Delta G_{obs}$ was calculated using

(6) $$\Delta \Delta G_{obs}=RTln\frac{EC_{50}}{EC_{50\left(bg\right)}}$$

where ${EC}_{50(bg)}$ is the ${EC}_{50}$ of the background (WT or E702Q) on which the mutation of interest was constructed. We have constructed the K588E and K645E mutations on the E702Q background to improve the expression of the constructs. $\Delta G_{obs}$ is equivalent to $\Delta G_{binding}$ when the affinities of all binding reactions are affected equally without affecting conformational transitions, which is a general property of linked equilibria.

To estimate the contribution of open states with various Ca^2+^ occupancy to the ensemble current, the I-V data for WT were fitted to a weighted sum of I-V curves corresponding to zero, one and two calcium ions bound at the binding site,

(7) $$I_{total}=iI_{0Ca}+jI_{1Ca}+kI_{2Ca}$$

where $I_{0Ca}$, $I_{1Ca}$ and $I_{2Ca}$ are in the form of Equation 2 with parameters from G644P at zero Ca^2+^, G644P/E654Q at saturating Ca^2+^ and G644P at saturating Ca^2+^ respectively. Since WT does not display noticeable basal activity, the contribution of current corresponding to zero occupancy was considered negligible and the $iI_{0Ca}$ term was omitted.

Data analysis was performed using Clampfit 10.6 (Molecular devices), Excel (Microsoft), and Prism 5 and 6 (GraphPad). Numerical calculations were performed using the NumPy package (http://www.numpy.org/). Data are presented as mean ± s.e.m. Fitted parameters are plotted and are reported as best-fit value ± 95% confidence interval.

### Poisson-Boltzmann calculations

The electrostatic potential along the pore, identified using Hole (Smart et al., 1996), was calculated by solving the linearized Poisson-Boltzmann equation in CHARMM (Im et al., 1998; Brooks et al., 1983) on a 240 Å x 240 Å x 260 Å grid (1 Å grid spacing) followed by focusing on a 160 Å x 160 Å x 160 Å grid (0.5 Å grid spacing). Partial protein charges were derived from the CHARMM36 all-hydrogen atom force field (MacKerell et al., 1998). Hydrogen positions were generated in CHARMM. *In silico* mutagenesis was carried out in CHARMM. The protein was assigned a dielectric constant ($\varepsilon_{r}$) of 2. The membrane was represented as a 35-Å-thick slab ($\varepsilon_{r}$= 2). A 5-Å-thick slab was included on each side of the membrane to account for the headgroup region ($\varepsilon_{r}$= 30). The bulk solvent on either side of the membrane and the solvent-filled conduit were represented as an aqueous medium ($\varepsilon_{r}$= 80) containing 150 mM mobile monovalent ions. Electrostatic calculations were carried out in the absence of an applied voltage. Although the structure of the protein used for calculations might not display a fully activated conformation, conformational changes are assumed to be small and would mainly affect the narrow neck region for which the dielectric constant is uncertain (Paulino et al., 2017a). The region most relevant for this study concerns the wide intracellular vestibule, which was assigned bulk-like dielectric properties. In calculations of mutants, positively charged residues were introduced as protonated histidines instead of arginines for modeling purposes.

## Additional files

Transparent reporting form

### Data availability

All data generated or analysed during this study are included in the manuscript.
